# Mechanical Properties and Synergistic Interfacial Interactions of ZnO Nanorod-Reinforced Polyamide–Imide Composites

**DOI:** 10.3390/polym15061522

**Published:** 2023-03-19

**Authors:** Dallas Kesler, Bhanuka P. Ariyawansa, Hemali Rathnayake

**Affiliations:** 1 Nanoscience Department, University of North Carolina Greensboro, Greensboro, NC 27412, USA; 2 Middle College, University of North Carolina Greensboro, Greensboro, NC 27412, USA

**Keywords:** polyimides, zinc oxide nanorods, nanofillers, polymer composites, nano-reinforced materials

## Abstract

Metal oxide nanoparticle -reinforced polymers have received considerable attention due to their favorable mechanical properties compared to neat materials. However, the effect of nanoscale reinforcements of the interface on the composites’ mechanical properties has not been investigated in-depth to reach their optimal performance in structural applications. Aiming at revealing the effect of synergistic interfacial interactions on the mechanical properties of polymer composites, using a nanoscale reinforcement, herein, a series of zinc oxide nanorod-reinforced polyamide–imide (PAI)/ZnO) composites were fabricated and their mechanical properties and viscoelastic responses were investigated. The composite prepared by reinforcing them with 5 wt % ZnO nanorods resulted in improved elastic modulus, stiffness, and hardness values by 32%, 14% and 35%, respectively, compared to neat polymer thin films. The viscoelastic dynamics of the composites revealed that there was an 11% increase in elastic wave speed in the composite, containing 5 wt % ZnO nanorods, indicating better response to high impacts. Delayed viscoelastic response decreased by 67% spatially and 51% temporally, with a corresponding decrease in the creep rate, for the 5 wt % ZnO nanorod- containing composite, evidencing its potential applicability in high strength lightweight structures. The improved mechanical properties with respect to the filler concentration evidence strong particle–polymer interfacial interactions, creating “chain-bound” clusters, providing clear reinforcement and polymer chain mobility retardation. However, hypervelocity impact testing revealed that all the composites’ films were vulnerable to hypervelocity impact, but the spallation region of the composite films reinforced with 2.5 wt % and 5 wt % ZnO nanorods exhibited a cellular-like matrix with shock-induced voids compared to a rather hardened spallation region with cracks in the neat film.

## 1. Introduction

Light, stiff, and strong materials capable of enduring a bearing load have become increasingly valuable for the design and construction of lightweight structural materials [1,2,3,4,5]. Solid materials such as engineered ceramics, metallic alloys, metal matrix composites, and ceramic matrix composites have been the standard for durable structures due to their high strength and stiffness, but they are not lightweight [6,7,8]. Materials such as polymers, elastomers, and conventional epoxy-based carbon-fiber-reinforced polymers (CFRPs) are lighter but susceptible to impact damage and elevated temperatures, and lack the required strength and stiffness compared to ceramic lattices. Additionally, the challenges in processing, large-scale manufacturing, and the cost-factor are a particularly serious problem of current state-of-the art nanocomposites for engineering applications. For successful use in a variety of applications, nanocomposites must become more affordable, readily available, reliable/reproducible, and repairable, and must exhibit equivalent or better properties than competing graphite/epoxy or metallic parts. The retention of mechanical properties of macroscale structures, accessible nanomanufacturing methods to permit the integration of functionalities, and the scale-up of manufacturing methods to enable the evaluation of larger components in relevant environments are also expected to be significant challenges. Thus, designing high-performance nanocomposites that are damage-tolerant lightweight, of a high strength and stiffness, and have tailorable multi-functionality for efficient hypervelocity impact (HVI) shielding, thermal management, and energy dissipation, with performance optimization at structural scales, are critical.

Polymer composites have been explored as possible high strength-to-mass ratio alternatives as light weight structural materials, especially in spacecrafts [9,10]. Since the dawn of the space age, aromatic polyimide (PI) has been used as a structural material in spacecrafts due to its high strength-to-mass ratio, and thermal and radiation stability. The presence of conjugated π cycles along the polymer backbone allows for a secondary π–π interaction induced structure with the nanofillers [11,12]. Despite these advantages, Verker et al. [13] demonstrated that PI suffers from poor atomic oxygen resistance, resulting in increased erosion. Additionally, PI is quite brittle, due to its rigid backbone, is poorly soluble in common solvents, and suffers from poor meltability, making processing a challenge [12]. Amide linkages have been added to the backbone of PI, yielding polyamide–imides (PAI), which improve the structural properties of the material as well as improving solubility and therefore processability [14,15,16].

The ability of small concentrations of nanofillers to enhance the mechanical properties, such as stiffness, hardness, and elastic modulus, of various polymer systems is well-established [17,18,19,20]. The high surface area-to-volume ratio of nanoparticles compared to that of their bulk equivalents means there is a greater surface for the particle to interact with, resulting in strong non-covalent interactions and a stronger material [18]. Equally, the relationship among these mechanical properties and the speed of elastic waves that propagate through a medium is well-established. The speed of elastic waves relates to hypervelocity impact damage; when the speed of elastic waves in a medium is overtaken by the speed of plastic waves due to collision, a shock forms, which causes significant damage to the material [21,22,23]. A filler material of particular interest is zinc oxide nanoparticles (ZnO NPs), owing to its unique properties such as thermal and mechanical stability at room temperature, good physical and chemical stability, environmental friendliness, abundant availability, and low cost. In the past literature, it has been shown that adding ZnO NPs as a filler material (2 wt % ZnO NPs of 50 nm in size) to a glass fiber network (50% glass fiber with 48% epoxy) improved impact strength [17]. Baghdadi et al. developed spherical ZnO NP epoxy resin blends and found that tensile strength was maximized by adding a 2.5 wt % ZnO nanofiller [24]. Additional research conducted by Wacharawichanant et al. has also confirmed the occurrence of similar effects on the properties of polyoxymethylene, with the maximum impact strength coming from composites containing approximately 1 wt % ZnO NPs [20]. 

Despite the use of ZnO nanoparticles as nanofillers, ZnO nanowires (NWs) have also been used to enhance the structural and mechanical properties of polymer composites [22,23,24,25]. For example, it has been demonstrated that vertically aligned ZnO NWs on the surface of carbon fiber led to a 113% increase in the interfacial shear strength of fiber-reinforced polymer composites [22]. Moreover, the length and diameter of ZnO NWs across the interface were considered as design parameters in tailoring interfacial strength, where interfacial shear strength can increase by three times [24]. The three-dimensional connectivity of a nanowire matrix that can act with high mechanical interlocking allows for better load transfer from the reinforcement phase (carbon fiber) to the polymer matrix. Thus, the nanowire interphase can offer a unique load transfer mechanism across the interface, providing better load bearing properties to the composites. Towards this goal, this study aims to demonstrate the impact of synergistic interactions of ZnO nanorods on the mechanical properties of a polyamide–imide (PAI) resin to produce mechanically robust polyamide–imide composites. A series of ZnO nanorod-reinforced PAI composites were prepared, their mechanical and viscoelastic properties, including creep and delayed viscoelastic response, were characterized via nanoindentation, and their thin films’ surface morphologies were characterized via SEM and AFM. Further, their mechanical strength was tested against hypervelocity impacts to utilize them as lightweight composites for space structures.

## 2. Materials and Methods

### 2.1. Materials

Zinc(II)chloride and dimethylformamide (DMF) were obtained from Fisher Scientific, USA. PAI resin, poly(trimellitic anhydride chloride-co-4,4′-methyldianiline) (PTMMDA), and n-methyl-2-pyrollidone (NMP) where obtained from Sigma Aldrich, USA. All chemicals were used without further purification, and NMP was stored in an air- and moisture-free environment. 

### 2.2. Instrumentation

Hitachi S-4800 FESEM was used for imaging the morphologies of zinc oxide nanorods and composite thin films. TI 950 TriboIndenter with TriboScan and the Triboview software, which include modulus mapping, Tribo TC, Tribo view and TriboImage (Hysitron, USA) long with a <0.5 μm 60° diamond cono-spherical tip, obtained from Hysitron, USA, was used for nanoindentation analysis, including AFM imaging. The hypervelocity impact testing experiments on ZnO nanorod-reinforced polymer composite films were conducted at the Marshall Space Flight Center (Huntsville, AL, USA) using the Microballistic Powder Gun (Marshall Space Flight Center, Huntsville, AL, USA) 

### 2.3. Synthesis of ZnO Nanorods

Following our previously published procedure by Davis et.al., ZnO nanorods were synthesized using sol-gel polymerization followed by solvent-driven self-assembly and crystal growth [26]. In a typical procedure, ZnCl_2_ (2.5 g, 18.3 mmol) was dissolved in anhydrous DMF (310 mL) with stirring and five minutes of sonication at room temperature. An aqueous solution of NaOH (3.7 g, 92.5 mmol) in water (56 mL) was added to the DMF solution while stirring at room temperature. The solution was stirred for ~15 min and then the temperature was raised to 80 °C and held for one hour. After an hour of stirring at 80 °C, stirring was stopped and the solution was kept static at 80 °C for 24 h. After 24 h, the resulting white powder was collected by centrifugation and the product was washed multiple times by resuspending it in water and repetitive centrifugation. The product collected in this manner was allowed to dry, and the morphology was characterized by SEM. The powder XRD obtained was matched with the simulated powder XRD to confirm the wurtzite structure of ZnO nanorods.

### 2.4. Preparation of Thin Films of ZnO Nanorods Reinforced PAI Composites

800 mg PTMMDA (Mw = 28,860 g/mol, 800 mg) was dissolved in anhydrous NMP (8 mL) by sonication (30 min). The desired amount of ZnO nanorods (20 mg, 40 mg, 80 mg, and 160 mg) was dispersed in NMP (8 mL) by several minutes of vortexing followed by 30 min of sonication. The PAI resin solution and ZnO NR suspension were combined and sonicated for another 30 min followed by overnight stirring in an inert atmosphere under a N_2_ environment. A copper block (5 cm × 5 cm × 6 mm mold cut) was covered with aluminum foil, forming a tray in the mold. The aluminum-lined mold was filled with the nanocomposite solution and heated to 120 °C, 150 °C, 180 °C, 195 °C, and 230 °C, being held at each temperature for an hour. The resulting composite on the aluminum foil was removed from the copper block and allowed to cool before imaging it via SEM.

### 2.5. Mechanical Property Measurements

Nanoindentation was used to obtain mechanical properties, such as stiffness, reduced elastic modulus, which is essentially equivalent to elastic modulus, but is measured using nanoindentation, and thus is referred as reduced elastic modulus, and hardness, creep analysis, and delayed viscoelastic response. For stiffness, reduced elastic modulus, and hardness, a sample film with an area of 0.5 cm^2^ was used from each composite composition. Using the Triboscan software, a 15 μm square region was defined on the sample thin film to obtain 16 indentations. A load function with a constant onloading rate from 0 to 150 μN over 5 s followed by a hold of 5 s maximum, and finally 5 s unloading at a constant rate was used. The delayed viscoelastic response analysis was conducted on these samples by maintaining constant onloading at a rate from 0 to 150 μN over 5 s followed by 5 s unloading at a constant rate. The constant loading and unloading process was applied to avoid the irregular plastic deformation and assume that the plastic deformation was minimal at the selected load. After each indentation, a topographical contact AFM image was taken with a scan rate of 1.00 Hz, tip velocity of 10.0 μm/sec, scan size of 5.0 μm, set point of 2.0 μN, and integral gain of 240.

### 2.6. Hypervelocity Impact Testing

Hypervelocity impact testing was conducted at the NASA Marshall Space Flight Center (MSFC) using Microballistic Powder Gun, which is designed to shoot polymeric beads to simulate raindrop impacts. Using customized beads, bead sizes of 3 mm (0.08 to 0.157 in) in diameter were used to simulate the more extreme raindrop sizes. This gun has very good impact accuracy at consistent velocities. An adjustable sample stage was used to vary the angle of attack and to allow multiple shots on one target. The beads were shot at a velocity range of 6130–6332 ft/s (Mach 5.5), which was measured using high-speed digital video. In a typical testing condition, 1–3 consecutive shots were fired while holding the sample at angle of 90°. The thin film sample size was 1 × 1 inches with a film thickness of 1 mm and was casted on flat-plate aluminum foil. The live digital video was collected for all the samples. 

## 3. Results

### 3.1. Morphology and Crsystallinity of ZnO Nanorods

**Figure 1:**
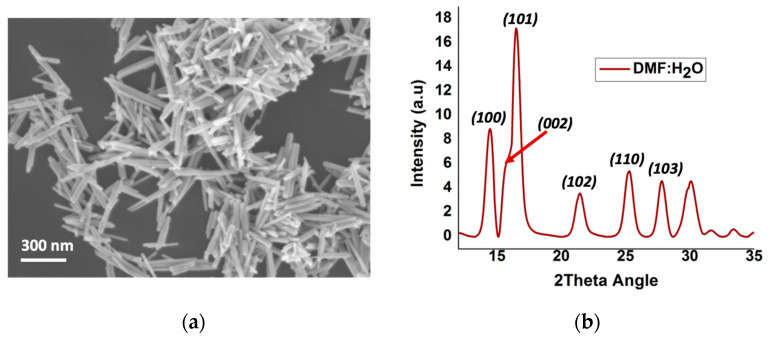
(**a**) SEM image of ZnO nanorods; (**b**) X-ray powder diffraction pattern of ZnO nanorods.

The morphology of the ZnO nanorods prepared in the water/DMF solution, following the previously published sol-gel synthesis method [27], exhibited needle like rods average dimensions of a 300 nm length and 30 nm diameter (Figure 1a). The X-ray powder diffraction pattern of the ZnO nanorods shown in Figure 1b were indexed to the hexagonal phase’s wurtzite crystal structure and was in good agreement with that in the published literature [26].

### 3.2. Dynamic Mechanical Properties of PAI Composites

The primary mechanical properties, which include stiffness (S), reduced elastic modulus (Er), and hardness (H), of different weight ratios of ZnO nanorod-reinforced polyamide–imide films were evaluated. As defined by Equations (1)–(3), these three mechanical properties rely on the depth of penetration by the tip of the indenter into the material. For instance, a decrease in the penetration depth at a constant load for composites with an increased filler concentration results in increased stiffness, reduced elastic modulus, and hardness.

(1) S=ΔP/ΔH

(2) $$E_{r}=\frac{S\sqrt{\left(\pi \right)}}{2\beta \sqrt{\left(A_{c}\right)}}$$

(3) $$H=\frac{p_{max}}{A_{c}}$$

where ∆P is the change in load at unloading and ∆H is the change in depth at unloading, $A_{c}=\pi h_{c}^{2}tan^{2}\left(\alpha \right)$, α is the semi apex angle of the tip, β is a geometrically dependent constant, which is equal to 1 for a cono-spherical tip, $h_{c}$ is the depth of the circle formed by the projection of the tip into the sample, and $p_{max}$ is the maximum applied load. 

**Figure 2:**
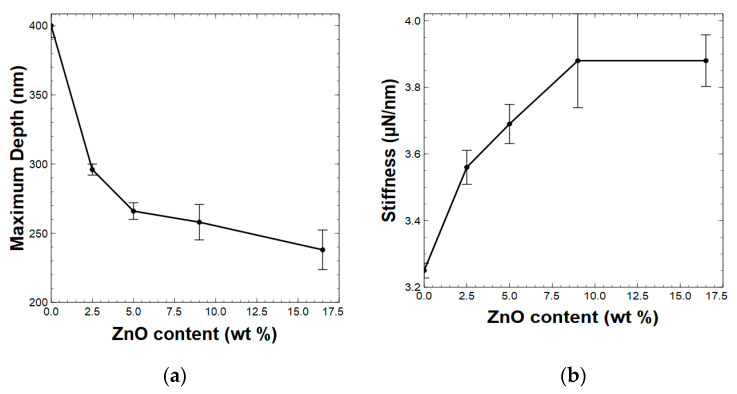
Distributions of (**a**) depth of penetration, and (**b**) stiffness of composites with a different wt % of ZnO nanorods [standard deviations are marked with vertical black lines].

Upon varying the ZnO nanorods’ filler concentration, the composite’s stiffness, reduced elastic modulus, and hardness were calculated using Equations (1)–(3). First, we mapped the relationship between the filler concentration, which ranged from a 0 wt % to 16.5 wt % concentration of ZnO nanorods, and the depth of penetration at a load of 150 μN. As seen in Figure 2a, the depth of penetration decreased as the ZnO nanorod weight% increased, suggesting that the interfacial interactions of polymer chains increase with respect to the weight% of ZnO nanorods. Additionally, the presence of a higher concentration of nanorods could affect the penetration difficulty, as a result of the filling of the free volume among the polymer chains by the nanorods. The changes in the depth of penetration of the composites further imply that changes in the dynamic behavior of the composites can be attributed to the polymer’s viscoelasticity. As expected, the reduced depth of penetration with respect to the filler concentration showed clear improvement in the composite’s stiffness (Figure 2b), where a linear increase (R^2^ = 0.93) in stiffness from 3.25 μN/nm at 0 wt % ZnO to 3.88 μN/nm at 9% ZnO was observed (Table S1). The stiffness remained unchanged after the filler concentration reached to 9 wt %, indicating that the interfacial interactions among the ZnO nanorods and polymer chains were maximized at a 9 wt % filler concentration.

**Figure 3:**
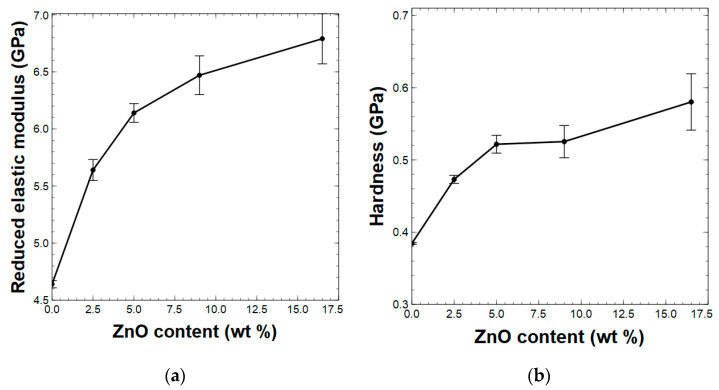
The distribution of **(a)** reduced elastic modulus, and (**b**) hardness of composites with a different wt % of ZnO nanorods [standard deviations are marked with vertical black lines].

The distribution of the reduced elastic modulus of the composites with respect to the filler concentration exhibited a gradual linear increase (R^2^ = 0.97) from 4.64 GPa for the neat polymer films to 6.14 GPa for the 5 wt % ZnO nanorod-reinforced composite film and continued to be maintained at 6 GPa (Figure 3a and Table S1) for higher filler concentrations. Similarly, the hardness of the composites increased linearly (R^2^ = 0.98) from 0.38 GPa for neat polymer films to 0.52 GPa for the 5 wt % filler concentration and above (Figure 3b and Table S1).

### 3.3. Morphologies of PAI Composites

**Figure 4:**
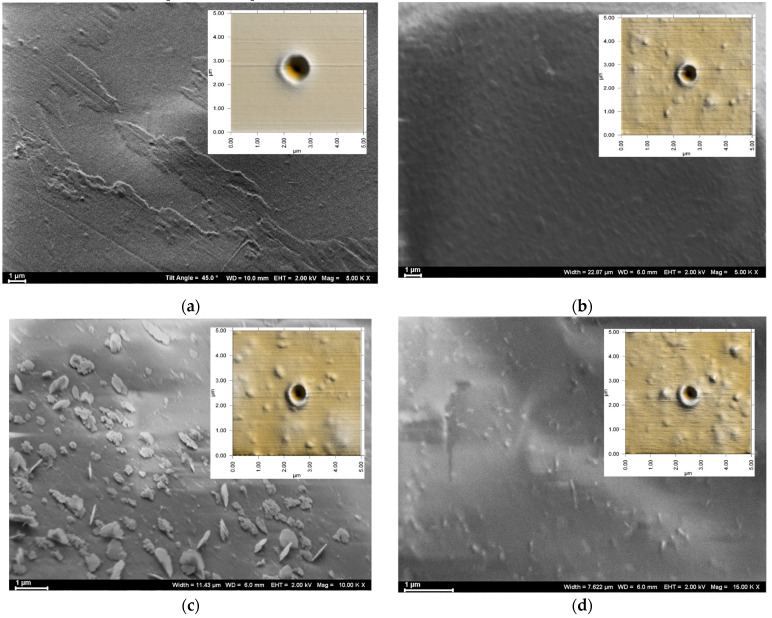
SEM images of composite thin films with a ZnO composition of: (**a**) neat polymer film; (**b**) 5 wt %; (**c**) 9 wt %; and (**d**) 16.5 wt %; inset—respective top-down AFM images of composite thin films after indentation.

The surface morphologies of the thin film composites were revealed by scanning electron microscopy (SEM) and are depicted in Figure 4. The neat thin film (Figure 4a) exhibited a rather smooth surface with randomly dispersed large flake-like aggregative assemblies. The composite thin films reinforced with 5 wt % ZnO nanorods (Figure 4b) showed homogeneously dispersed clusters, which were embedded within the polymer matrix. In the composite films with a higher concentration of ZnO nanorods, aggregative clusters were larger in size and more abundant. For instance, the composite with 9 wt % ZnO resulted in poorly dispersed films (Figure 4c) with large clusters, which were aggregated on the surface rather than embedded within the polymer matrix, whereas the 16.5 wt % ZnO nanorod-reinforced composite films exhibited large clusters, which were embedded within the polymer matrix. From the SEM images, it is evidenced that 5 wt % ZnO nanorod-reinforced PAI polymer composites show better dispersibility and a better balance between the filler concentration and polymer matrix, correlating with its mechanical properties. 

Supporting the SEM images, top-down AFM images and surface roughness profile images of each composite were also obtained after the indentation and are shown in Figure 4 (inset) and Figure S1, respectively. As summarized in Table S2, the surface roughness of each composite film obtained from the AFM depth profile images agrees with the filler concentration and speaks to the surface morphologies revealed from the SEM images and top-down AFM images. With the increase in the filler concentration, there was a significant increase in the surface roughness of the composites, in which the 5 wt % ZnO nanorod-reinforced composite exhibited ~4 nm surface roughness whereas the 9 wt % and 16.5 wt % ZnO nanorod-reinforced composites possessed surface roughness values of ~14 nm, suggesting the favorable balance in interfacial interactions between ZnO nanorods and polymer chains under a low filler concentration vs. the presence of stronger interparticle interactions among ZnO nanorods at a higher filler concentration compared to the presence of particle–polymer chain interactions. Similar findings have been previously reported by many researchers where the aggregative cluster formations were observed at a higher filler concentration of ZnO nanoparticles in different polymer matrices [17,20,24,27].

### 3.4. Rule of Mixture and Modified Rule of Mixture Model

To gain insight into the effect of ZnO nanorod-reinforcement on the composites’ elastic moduli, we applied two known rule-of-mixture models (ROMs) and a modified rule-of-mixture model (MROMs). To predict the upper and lower boundaries of the elastic modulus of the PAI composites, we applied Voigt and Reuss equations (Equations (4) and (5)) and compared them with the experimentally reduced elastic moduli of the composites. The models are essentially weighted means of the elastic modulus, or the inverse of the elastic modulus of the matrix and filler.

(4) $$E_{c}=V_{m}E_{m}+\left(1-V_{m}\right)E_{f}$$

(5) $$E_{c}^{-1}=V_{m}E_{m}^{-1}+\left(1-V_{m}\right)E_{f}^{-1}$$

where *E_c_*, *E_m_*, and *E_f_* are the elastic modulus of the composite, matrix, and filler, respectively, and *V_m_* is the volume fraction of the matrix, which is 0.45 for polyamide–imides. The *E_f_* of ZnO nanorods was taken from the prior literature, [28] which reported the elastic modulus for ZnO nanorods of a diameter of 36 nm, and *E_f_* = 155 GPa. For *E_m_*, we used the experimentally measured reduced elastic modulus of neat polymer thin films, which is 4.64 GPa (Table S1). 

**Figure 5:**
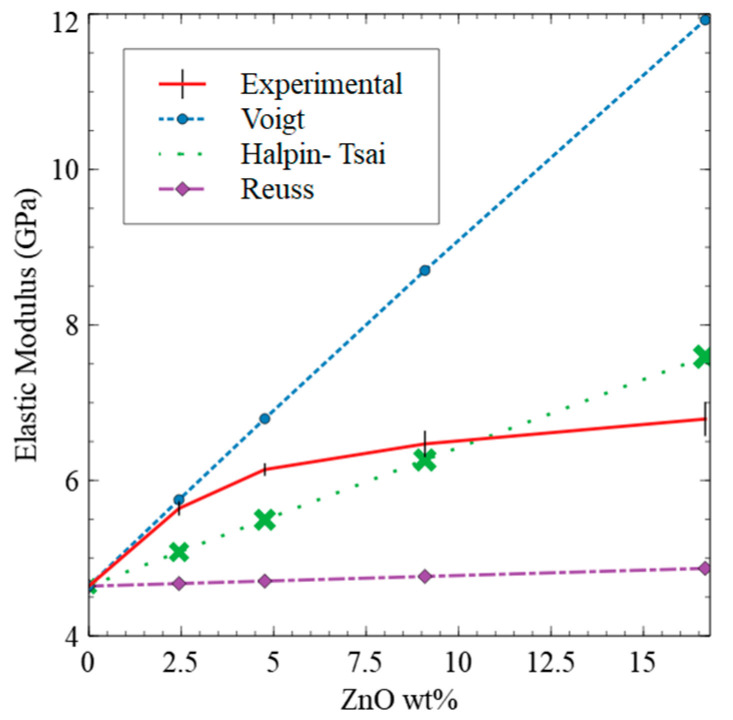
The comparison of experimental reduced elastic modulus with predicted elastic modulus by ROM and MROM models.

As depicted in Figure 5a, the elastic modulus of ZnO nanorod-reinforced composites varied with respect to the weight% of the ZnO nanorods and lay in between the Voigt and Reuss models for a filler composition higher than a 2.5 wt % concentration of ZnO nanorods. The Voigt model is acceptable to predict the elastic moduli of the composites with a ZnO nanorod concentration of up to 2.5 wt % and follows the Voigt approximation, which predicts the upper-bound elastic modulus of a composite, assuming that load is applied parallel to the filler; it is called the longitudinal stress. However, with the increase in the filler concentration, the elastic modulus of the composite deviates from the Voigt model and tends to follow the Reuss model where we observed a steady linear increase, starting at a filler concentration from 5 wt % to 16.5 wt %. Thus, the elastic moduli of composites with a higher filler concentration follows the Reuss approximation, which predicts the lower-bound elastic modulus based on the assumption that load is applied perpendicular to the filler; it is the called transverse stress [29].

Considering the aspect ratio of the filler in addition to considering the volume fraction of the matrix and the elastic modulus of the filler and matrix, we applied a MROM-Halpin–Tsai model, which is described by the Halpin–Tsai equation (Equation (6)). Unlike the Voigt and Reuss approximations, the Halpin–Tsai one provides a single value for the prediction of elastic modulus, not an upper and lower bound. As depicted in Figure 5, the experimental elastic moduli were higher than the predicted elastic moduli by the Halpin–Tsai model. This could be due to the strong interparticle interactions as a result of the high surface-to-volume ratio of the nanofiller. The Halpin–Tsai model is unable to account for the nanoscale effects of filler and thus underestimates the response of added nanofiller at these concentrations. However, beyond a 9 wt % ZnO nanorod concentration, the Halpin–Tsai model overestimates elastic modulus. Overall, predictions made from ROM models and the MROM model convey that a higher loads of ZnO nanorods have a lesser impact on enhancing the elastic modulus of the composites. This is because the addition of more ZnO can only contribute to load transfer by flow restriction.

(6) $$E_{c}=\frac{1+\eta \xi V_{f}}{1-\eta V_{f}}E_{m}$$

$$\mathrm{Where},\;\eta =\frac{E_{f}/E_{m}-1}{E_{f}/E_{m}+\xi }\;\mathrm{and}\;\xi =2\times \frac{l}{d};$$

*E_c_*, *E_m_*, and *E_f_* are the elastic modulus of the composite, matrix, and filler, respectively, and *V_m_* (0.45) and *V_f_* (0.32) is the volume fraction of the matrix and filler, respectively [30,31]. For *E_c_*, experimental values were used (Table S1), *E_m_* = 4.64 GPa, and *E_f_* = 155 GPa.

### 3.5. Viscoelastic Properties of PAI Composites

The elastic wave speed, creep, and delayed viscoelastic response of a composite provide insight to the susceptibility to hyper velocity impacts. Thus, we studied the viscoelastic properties of ZnO nanorod-reinforced PAI composites and correlated the results with the SEM images of composites obtained after they were subjected to hypervelocity impact testing. As shockwaves are major contributors to destruction from hypervelocity impacts, and as they occur when plastic waves in a medium travel faster than elastic waves do, we first calculated the elastic wave of the speed of each composite by applying Equation (7), which describes the speed of elastic waves in a medium [23].

(7) $$C_{o}=\sqrt{\frac{E\left(1-\nu \right)}{\rho \left(1+\nu \right)\left(1-2\nu \right)}}$$

where *C_o_* is the elastic wave speed, *E* is the elastic modulus, and ν is the Poisson’s ratio. To determine the Poisson’s ratio, a rule-of-mixture calculation was performed, using the weighted sum of the Poisson’s ratio of the matrix and filler. The Poisson’s ratio of the ZnO was set to 0.32, as determined in the prior literature [31], and the Poisson’s ratio for the polyamide–imide was set to 0.45 [30].

**Figure 6:**
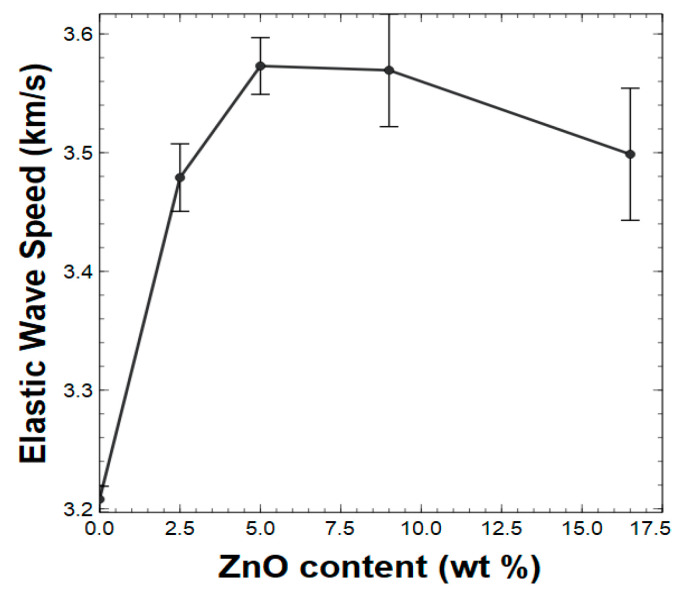
The distribution of elastic wave speed of composites with respect to the different wt % values of ZnO nanorods [standard deviations are marked with vertical black lines].

As summarized in Table S3, the neat polymer film’s elastic wave speed was calculated as 3.21 km/s, which increased by 11% for the composites containing up to a concentration of 9 wt % ZnO nanorods, with a slight decrease for the composite with the concentration of 16.5 wt % ZnO nanorods. At a higher concentration of ZnO nanorods, there was no significant change in elastic wave speed. The changes in elastic wave speed can be explained by the “bridging chain” cluster formation which leads to density changes under a larger filler weight. As ZnO nanorod content increased, so did the density of the material. For small amounts of filler concentration (2.5 wt % and 5 wt %), there was a significant increase in elastic modulus, offsetting the increase in density so that the elastic wave speed was improved. Figure 6 depicts the distribution of the elastic wave speed of the composites with respect to the wt % of the ZnO nanorods.

**Figure 7:**
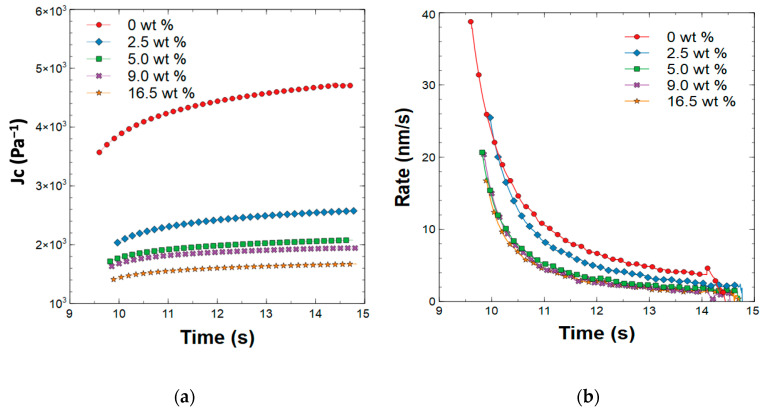
The creep behavior plots as a function of time for (**a)** displacement (J_c_) during 5 s hold under 150 μN load, and (**b**) rate of displacement during hold.

To elucidate creep behavior, the composites were subjected to a 5 s hold under a 150 μN load, the displacement was measured as a function of time and its derivative was taken to generate a creep rate curve, which is depicted in Figure 7. Compared with the neat polymer film’s penetration depth, the penetration depths of the composites with different ZnO nanorod weight compositions decreased with increased filler weight. However, the rate of penetration was minimal for the composites with a filler concentration higher than 5 wt %.

**Figure 8:**
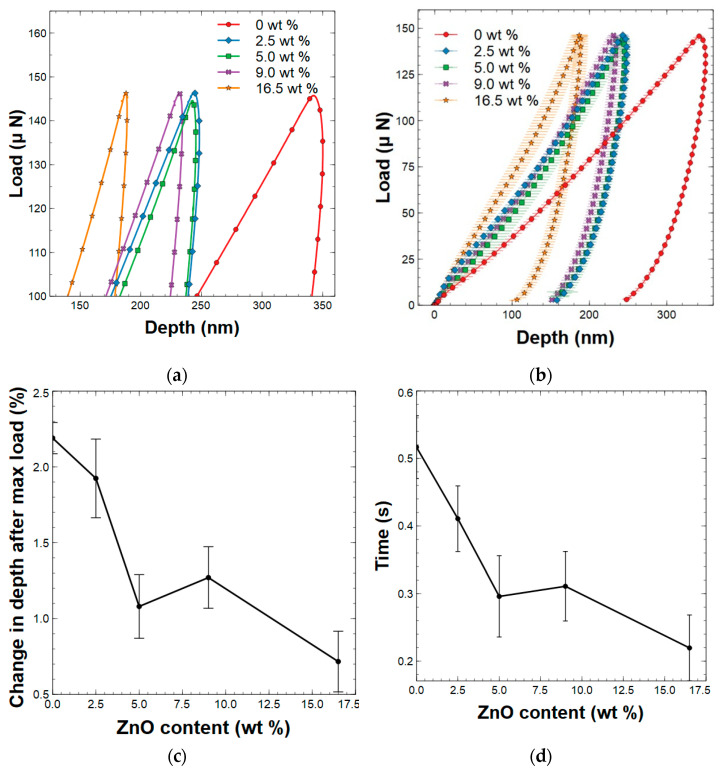
(**a**) Curvature in load displacement curve around the point of initial unloading, (**b**) Average load displacement with no hold at maximum load, (**c**) depth displacement after maximum load, and (**d**) viscoelastic response time with respect to wt % of the ZnO nanorods [standard deviations are marked with vertical black lines].

In the pursuit of understanding the delayed viscoelastic response, a nanoindentation experiment with no hold at the maximum load (150 μN) was performed and the load displacement curves are shown in Figure 8a,b. It is noted that neat PAI films, and 2.5 wt % and 5 wt % ZnO nanorod-reinforced composite films, exhibited the continued penetration after the point of maximum load. For the neat PAI films, as stress was applied to the polymer, the weak intermolecular π–π interactions were broken, leading to the movement of the polymer chains. Once the stress started to dissipate, the overall structure of the polymer was still quite weak, as it took time for the intermolecular interactions to recommence. Therefore, polymer chains would have continued to move due to whatever residual stress it still experienced. For the ZnO nanorod-reinforced polymer composites, the ZnO nanorods could both restrict polymer movement as well as absorb some of the stress. Therefore, the composite materials were less susceptible to continued deformation.

Temporal and spatial calculations were performed to quantify the delayed viscoelastic response. For temporal calculations, the time from the maximum applied load to the maximum depth was determined. For spatial calculations, the amount of penetration after the point of the maximum applied load was determined. Table S4 and Figure 8c,d shows the quantified delayed viscoelastic response of these PAI/ZnO nanorod composites. The 5 wt % and 16.5 wt % concentrations of ZnO nanorod-reinforced composites showed the best ability to attest to the delayed viscoelastic response. Given its poor elastic wave speed and mechanical properties, the ZnO nanorod-reinforced PAI composite, containing a concentration of 16.5 wt % ZnO nanorods was a poor material despite its ability to resist a delayed viscoelastic response, because of large aggregative clusters limiting strong interactions between nanorods and polymer chains. Additionally, these calculations show that the addition of 5 wt % ZnO nanorods greatly reduced the degree of the continued deformation of the composite, due to strong interfacial interactions between the polymer and the filler.

### 3.6. Evaluation of the Hypervelocity Impact’s Damage to the Composites

**Figure 9:**
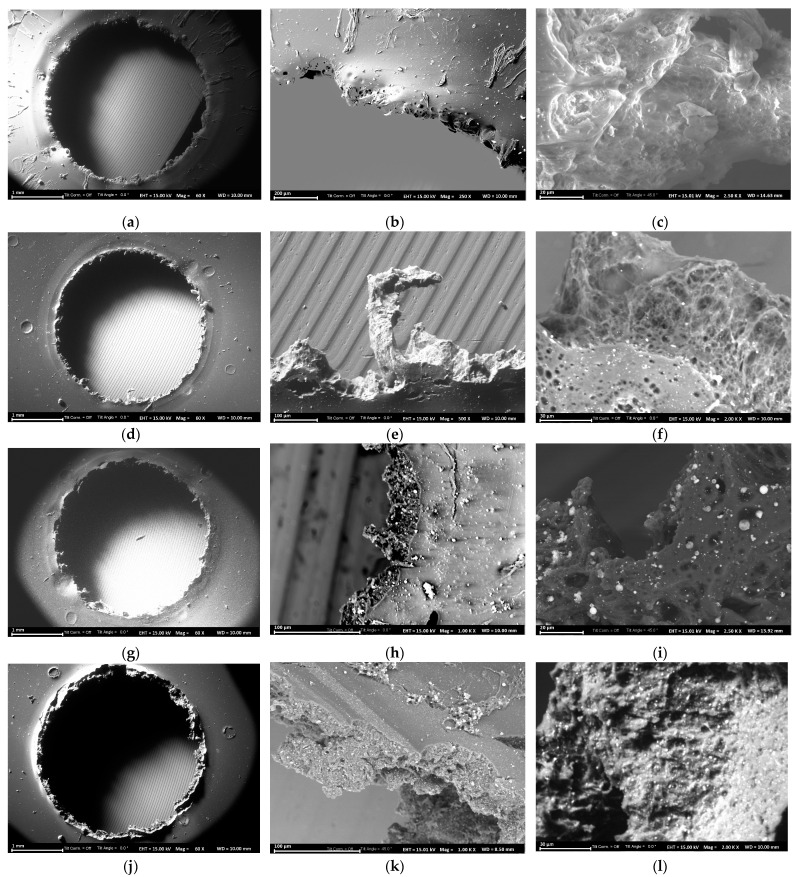
SEM images of (**a**–**c**) neat PAI film; (**d**–**f**) 2.5 wt % ZnO-reinforced PAI film; (**g**–**i**) 5 wt % ZnO-reinforced PAI film; and (**j**–**l**) 9.5 wt % ZnO-reinforced PAI film after hypervelocity impact testing.

To use PAI–ZnO nanorod composites as potential space shielding structures, initial studies on the hypervelocity impact’s damage of the composites were conducted. The material damage was evaluated by examining the morphology around the crater and the spallation damage of each composite. The morphologies of the films around the crater were imaged using SEM and are depicted in Figure 9.

Transitioning from neat PAI film to different a wt% of the ZnO nanorod-reinforced composite films, there were no noticeable changes in the crater size (Figure 9a,d,g,j) and all the films were damaged by the impact, resulting in a crater diameter of 3.5 mm. However, the morphology of the film’s spallation region was different in each composite compared to the crater edge of the neat polymer film. The spallation region of the composite films reinforced with 2.5 wt % and 5 wt % ZnO nanorods exhibited a cellular-like matrix with shock-induced voids (Figure 9f,i) compared to a rather hardened spallation region with non-cellular voids in the neat film (Figure 9c). The spallation region of the 9.5 wt % ZnO nanorod-reinforced PAI film showed a rather deformed non-cellular matrix, and there was no indication of shock-induced voids. The improved elastic wave speed in the composites compared to the elastic wave speed of the neat polymer films could have been the reason for the cellular-like matrix with shock-induced voids in the spallation region of the low filler concentration composites. With the aggregation occurring in the ZnO nanorod-reinforced composites containing 9 wt % and 16.5 wt % ZnO nanorods, the meager improvement in elastic modulus is insufficient to compensate for the increase in density, and thus the elastic wave speed was not improved, resulting in a rather deformed non-cellular matrix with larger cracks instead of shock-induced voids upon subjection to the hypervelocity impact.

## 4. Discussion

**Figure 10:**
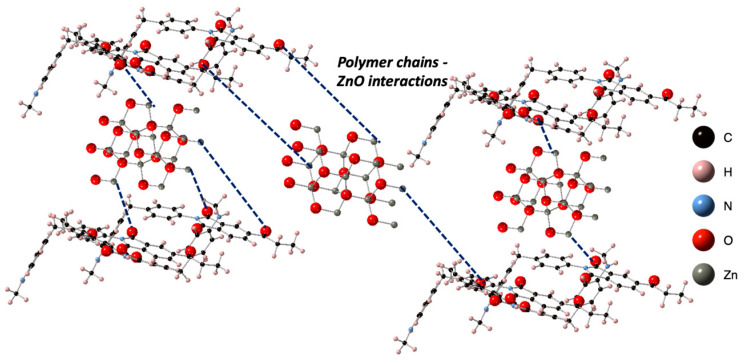
The schematic representation of PAI polymer chain–ZnO nanorod interactions, creating “bridging chains” of reinforcement and causing polymer chain mobility retardation.

The changes in mechanical properties with respect to the wt % of ZnO nanorod concentration can be explain by considering the possible interfacial interactions between the facets of ZnO nanorods and PAI polymer chains. Typically, the shape and size of nanomaterials contribute to the largest interfacial element surface and the shortest interelement distance when individually dispersed [32]. Therefore, they can induce the larger area of the surrounding polymer matrix to create polymer–particle interactions, providing clear reinforcement and causing polymer chain mobility retardation [32]. In our case, the metal cation nodes of the ZnO nanorods non-covalently interacted with the carbonyl functional groups in amide moieties in the PAI chains, creating so-called “bridging chains”, [33,34,35] which interact with multiple ZnO nanorods, yielding nanorods in chain-bound clusters (Figure 10). These clusters could behave as independent entities dispersed in a polymer matrix, creating a hierarchical structure that contributed to the final properties of the composites through element−polymer interfacial interactions and an intra-element nanorod−polymer-bridged nanostructure and provided intrinsic stiffness to the cluster. As stress was applied to the PAI matrix, some of this stress was transferred to these clusters as well as through flow restriction of the PAI by the nano-reinforcement caused by randomly oriented nanorods [36]. The result of this was that the effective stress on the matrix was only a fraction of the actual stress on the system, and thus it better resisted deformation than the neat polymer films did. Given the ZnO nanorod’s relatively high stiffness, the stress transferred to it by the PAI caused essentially no deformation of the ZnO nanorods.

The variation in surface roughness with respect to the filler concentration is a clear indication of how interfacial surface interactions between particle and polymer chains, and interelement distance between particles or particles and polymer chains affect the dispersibility of filler within the polymer matrix. For instance, at lower filler concentrations, interfacial surface interactions between nanorods and polymer chains are more favorable than the particle–particle interactions as interparticle distance is larger, forming a stable ZnO nanorod-reinforced polymer network, allowing for load transfer from PAI to ZnO nanorods. At a higher filler concentration, interparticle distance is smaller than the particle–polymer interfacial distance, resulting in larger aggregates of ZnO nanorods, limiting the favorable interactions with polymer chains. This interplay between the interfacial surface interactions and the interfacial distance among the particles and polymer chains has also been previously explained by Bindu et al. [18]—as the ZnO nanorod concentration increases, interparticle distance decreases until the attractive force between ZnO nanorods is greater than that between the polymer and ZnO, thus leading to aggregation. The result of this aggregation is a composite with a lower effective ZnO nanorod concentration than its actual concentration because a large amount of ZnO nanorods is trapped inside the aggregates and is unable to contribute to the enhancement of the mechanical properties of the composite. This speaks to our results in terms of the improvement in mechanical properties, where we observed minimal improvement in stiffness, reduced elastic modulus, and hardness of the composites with a higher wt % of ZnO nanorod concentrations (i.e., at 9 wt % and 16.5 wt %). Comparing the experimentally reduced elastic modulus values to the values given by the Voigt and Reuss upper and lower bounds as well as by the Halpin–Tsai approximation further supports the theory that load transfer and ZnO interaction with the polymer enhances the elastic modulus of the composites, while aggregation diminishes the efficacy of adding additional ZnO beyond 5 wt %.

## 5. Conclusions

Polyamide–imide composites reinforced with ZnO nanorods have been developed, and the role of synergistic interfacial interactions of ZnO nanorods on their mechanical properties has been investigated. We have demonstrated that reinforcing PAI with a lower wt % of ZnO nanorods, with the dimensions of a diameter of 30 nm and a length of 300 nm, improved the mechanical properties and viscoelastic response compared to the neat polymer’s mechanical and viscoelastic properties. Our findings of rule-of-mixture and modified rule-of-mixture models support the homogenous dispersion of nanorods within the polymer matrix, and the viscoelastic response analysis supports that the interfacial interactions between the polymer chains and the nanofillers, creating optimum nanoscale reinforcement via “bridging chain” clusters, are essential to produce lightweight, multifunctional, and high-performance polyamide–imide nanocomposites.

## Data Availability

Not applicable.

## Supplementary Materials

The following supporting information can be downloaded at: https://www.mdpi.com/article/10.3390/polym15061522/s1; Figure S1: Profile AMF images of composites showing surface roughness after post indentation—(a) Neat PMR film, and composite thin films with (b) 2.5 wt %, (c) 5 wt %, (d) 9.0 wt %, and (e) 16.5 wt % ZnO nanorods; Table S1: Mechanical properties of ZnO nanorods-reinforced PAI composites. AFM; Table S2: Approximate surface roughness of each composite measured from profile AFM, Table S3: Elastic wave speed of PAI/ZnO composites, and Table S4: The quantified %change in depth of PAI/ZnO nanorods composites with respect to the time from maximum load to maximum depth.
