# Supplementary material for: Mechanical Properties and Synergistic Interfacial Interactions of ZnO Nanorod-Reinforced Polyamide–Imide Composites
Source: Polymers (Basel). 2023 Mar 19;15(6):1522. doi: 10.3390/polym15061522 (PMC10055968; doi:10.3390/polym15061522)
Supplement: Supplementary file 1 [file polymers-15-01522-s001.zip › polymers-2235531-supplementary.pdf]

Supporting Information

Dynamic Mechanical Properties and Synergistic Interfacial Interactions of ZnO Nanorods Reinforced Polyamide Composites

Dallas Kesler,[1] Bhanuka P. Ariyawansa,[2] and Hemali Rathnayake [1,*]

Table S1. Mechanical properties of ZnO nanorods-reinforced PAI composites.

% Weight of ZnO nanorods (%wt)	Average maximum depth ± SD (nm)	Stiffness ± SD (µN/nm)	Reduced Elastic Modulus ± SD (GPa)	Hardness ± SD (GPa)
None	400 ± 8.50	3.25 ± 0.02	4.64 ± 0.03	0.384 ± 0.001
2.5	296 ± 4.00	3.56 ± 0.05	5.64 ± 0.09	0.473 ± 0.006
5.0	266 ± 6.00	3.69 ± 0.06	6.14 ± 0.08	0.522 ± 0.010
9.0	256 ± 12.8	3.88 ± 0.10	6.47 ± 0.20	0.525 ± 0.020
16.5	238 ± 14.3	3.88 ± 0.08	6.79 ± 0.20	0.580 ± 0.040

*R2 for stiffness, reduced elastic modulus, and hardness are at the level of 93%, 97%, and 98% confidence level, respectively.

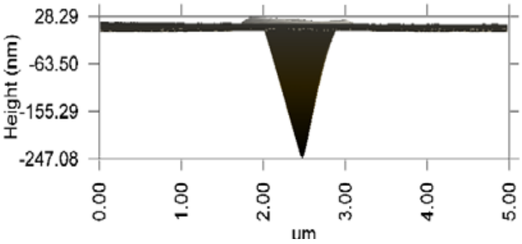

(**a**)

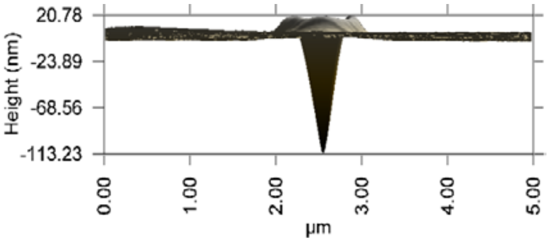

(**b**)

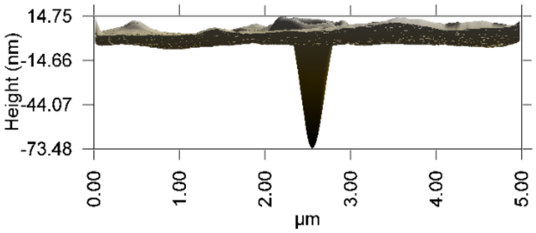

(c)

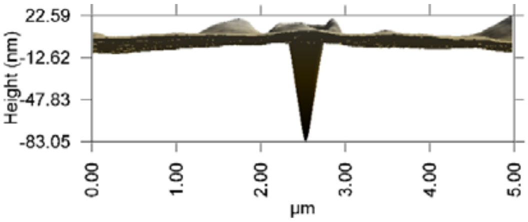

(d)

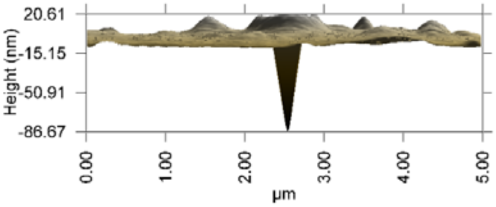

(e)

Figure S1: Profile AMF images of composites showing surface roughness after post indentation – (a) Neat PMR film, and composite thin films with: (b) 2.5 wt %, (c) 5 wt %, (d) 9.0 wt %, and (e) 16.5 wt% of ZnO nanorods.

Table S2: Approximate surface roughness of each composite measured from profile AFM

ZnO Concentration (wt %)	Approximate Surface Roughness (nm)
0	0
2.5	0
5.0	4
9.0	13
16.5	14

Table S3: Elastic wave speed of PAI/ZnO composites

**Wt% of ZnO nanorods**	**Speed of Elastic Wave (km/s)±SD**	**Percent change from neat polymer to composites**
0	3.21±0.01	0.00
2.5	3.48±0.03	8.45
5.0	3.57±0.02	11.4
9.0	3.57±0.05	11.3
16.5	3.50±0.06	9.06

Table S4: The quantified %change in depth of PAI/ZnO nanorods composites with respect to the time from maximum load to maximum depth

ZnO wt % in composite	Percent change in depth	Time (s) from maximum load to maximum depth
0	2.2	0.51
2.44	1.92	0.41
4.76	1.08	0.30
9.09	1.27	0.31
16.67	0.73	0.22
